# A Multifunctional Flexible Sensor Based on a Hybrid Microstructured Functional Layer

**DOI:** 10.3390/mi17080898

**Published:** 2026-07-27

**Authors:** Jianxiang Wang, Hongbin Chen, Yu Zhang, Jingmei Li, Zhengyun Zhong, Yue Li, Yanzhang Yang, Man Zhang, Meng Zhang, Wu Zhang, Lip Ket Chin

**Affiliations:** 1 School of Physics and Material Science, Guangzhou University, Guangzhou 510006, China; wangjianxiang@e.gzhu.edu.cn (J.W.); 2112119043@e.gzhu.edu.cn (H.C.); yu.zhang@e.gzhu.edu.cn (Y.Z.); 2 School of Electronics and Communication Engineering, Guangzhou University, Guangzhou 510006, China; 32405100110@e.gzhu.edu.cn (J.L.); 32419500043@e.gzhu.edu.cn (Z.Z.); manzhang401@gzhu.edu.cn (M.Z.); zhangmeng@gzhu.edu.cn (M.Z.); 3 Department of Electrical and Electronic Engineering, The Hong Kong Polytechnic University, Hong Kong SAR 999077, China; yue-li.li@connect.polyu.hk; 4 Guanghua Institute of Sci-Tech (Guangdong) Co., Ltd., Guangzhou 510006, China; yangyanzhang@ghtech.com

**Keywords:** flexible sensors, hybrid microstructure, pressure sensor

## Abstract

Flexible capacitive sensors for electronic skins and soft robotic systems are expected to provide not only high-pressure sensitivity but also multifunctional sensing capabilities. However, conventional dielectric layer designs often suffer from a trade-off among multiple functionalities. To address this challenge, we developed a flexible sensor featuring a hybrid microstructured functional layer for pressure sensing, distance monitoring, and material identification. The functional layer was a polydimethylsiloxane (PDMS) film embedded with micro-sized sugar particles and patterned with microstructures on its surface. The pressure-sensing performance, such as pressing sensitivity, response time, and hysteresis, was first evaluated. The pressure sensitivity reached 3.55 × 10^−2^ kPa^−1^ at an applied force of 1 N, which is significantly greater than that of the sensor using either a flat PDMS layer or a PDMS film embedded solely with sugar particles. The hybrid microstructured sensor also exhibited superior performance in terms of hysteresis and repeatability. Moreover, the sensor was shown to measure the distance to an object with a sensitivity of 0.023 mm^−1^. Furthermore, the robust identification of materials with different permittivities was demonstrated using the flexible sensor. Given its multifunctional, non-contact, and high-sensitivity capabilities, this flexible sensor holds significant potential for integration into advanced electronic skins, intelligent soft robotics for tactile object sorting, and human–-machine interfaces.

## 1. Introduction

Flexible electronics have emerged as a rapidly growing research field owing to their distinctive advantages, such as flexibility, lightweight, biocompatibility, and conformal contact, which enable extensive applications in electronic skin, wearable healthcare, soft robotics, and human–machine interfaces [1,2,3,4,5,6]. As a core component of flexible electronic systems, flexible pressure sensors convert external mechanical stimuli into measurable electrical signals and have become essential for detecting human physiological signals, enabling robot tactile perception, and monitoring motion [7,8]. Among various transduction mechanisms, capacitive flexible pressure sensors are particularly appealing because of their simple structure, low power consumption, low temperature/humidity drift, stable signal output, and ease of large-area integration [9,10,11].

The key performance indicators of capacitive pressure sensors, such as sensitivity, response time, working range, and hysteresis, are substantially determined by the structural and material properties of the functional layer [12,13]. Over the past decade, the microstructural engineering of functional layers has been widely acknowledged as the most effective strategy for enhancing sensitivity. Pioneering research by Mannsfeld et al. demonstrated that microstructured rubber functional layers could significantly enhance the sensitivity of flexible capacitive sensors [14]. Since then, numerous surface micropatterns, such as micropyramids, micropillars, microdomes, wrinkles, and gradient textures, have been developed to reduce the effective elastic modulus and amplify capacitance changes under low pressure [15,16,17,18,19,20]. These surface-patterned functional layers notably improve low-pressure sensitivity but often exhibit rapid saturation at medium-to-high pressure and limited linearity [21,22,23,24].

Simultaneously, internal particle doping and porous engineering have been investigated as alternative methods to enhance sensitivity without complex surface patterning. By incorporating high-dielectric-constant fillers, conductive nanomaterials, or sacrificial templates, such as salt, into polydimethylsiloxane (PDMS) matrices, researchers have successfully increased deformability and effective permittivity during compression [25,26,27,28]. For example, porous PDMS dielectrics exhibit an enhanced pressure response due to the gradual closure of internal voids [29,30,31]. However, sensors relying solely on internal doping or porosity often exhibit increased hysteresis and poor consistency [32,33]. Motivated by these challenges, this work presents a multifunctional, flexible capacitive sensor that enhances pressure sensitivity and extends its sensing capabilities beyond conventional contact pressure detection. By addressing the drawbacks of isolated approaches, the proposed hybrid dielectric layer synergistically combines embedded sugar particles with a serpentine microstructure. This dual-strategy architecture simultaneously improves pressure-sensing performance and enables non-contact distance monitoring and material identification. Such versatile sensing capability is highly attractive for wearable electronics, electronic skins, intelligent soft robotics, and human–-machine interfaces, where both contact and proximity information are essential.

**Figure 1:**
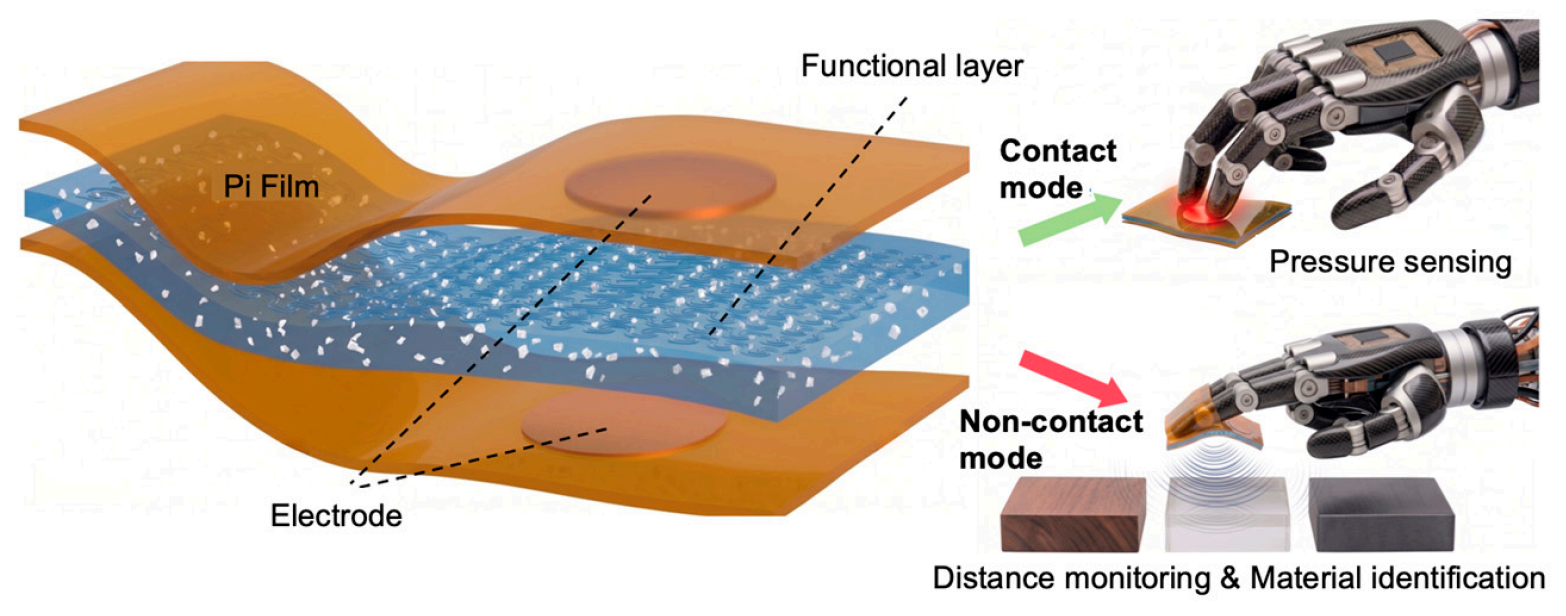
Multifunctional flexible sensor based on a hybrid microstructured functional layer.

In this study, we designed a flexible electronic sensor comprising a hybrid-structured functional PDMS layer, as shown in Figure 1. The functional layer was embedded with micro-sized sugar particles and patterned with a surface microstructure to achieve high pressure sensitivity. Other sensing performance metrics, including response time, detection limit, and hysteresis, were investigated. Additionally, we demonstrated that the sensor can be effectively employed to monitor distance changes and identify materials with different permittivities under non-contact conditions.

## 2. Materials and Methods

**Figure 2:**
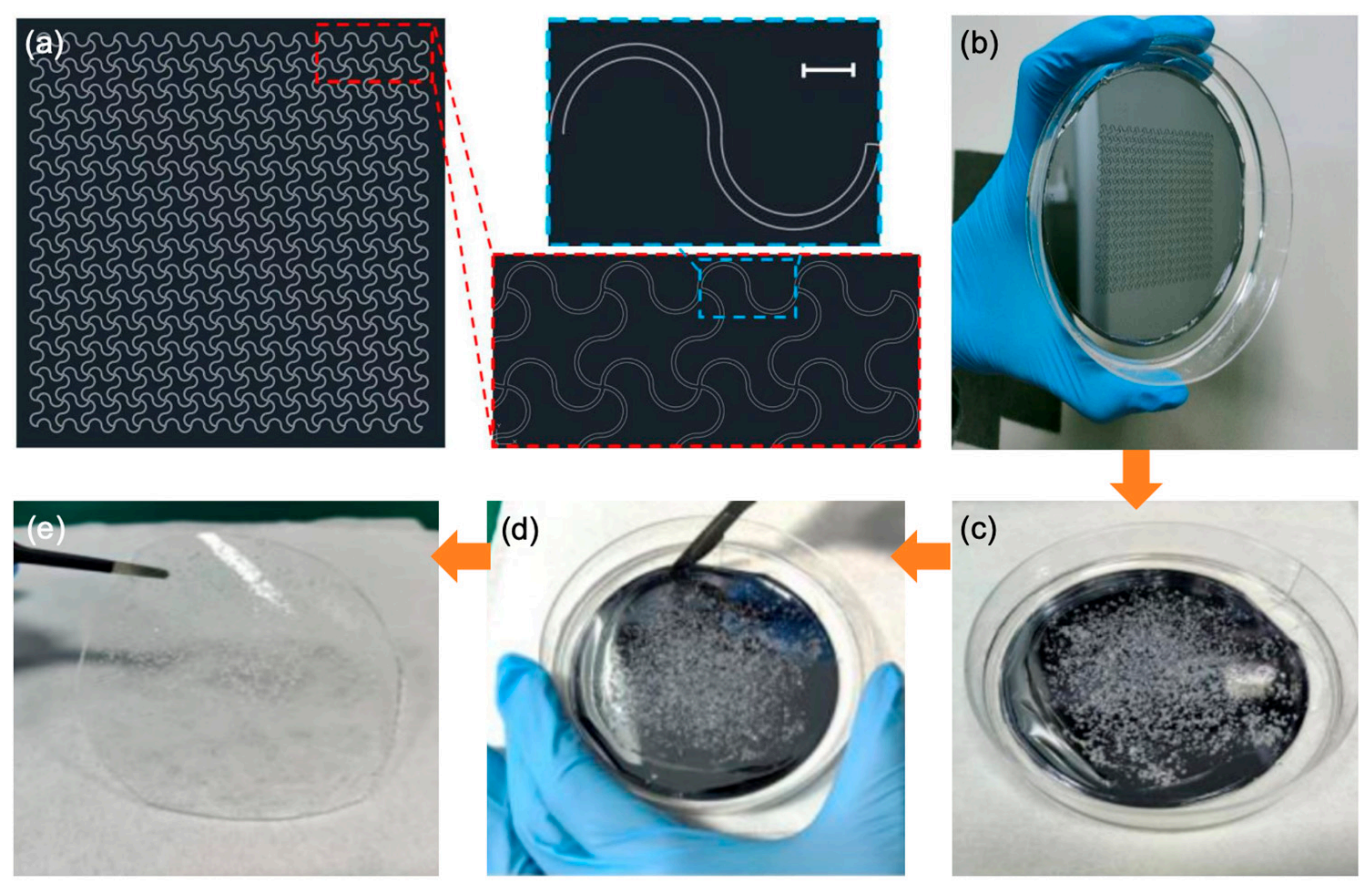
(**a**) Serpentine structured layout (scale: 600 μm); (**b**) serpentine structured mold on silicon wafer; (**c**) liquid PDMS with sugar particles on the mold; (**d**) cured PDMS with sugar particles on the mold; (**e**) PDMS layer with sugar particles peeled off from the mold.

The microstructure of the dielectric functional layer, as shown in Figure 2a, features a 15 × 15 array of serpentine elements, each comprising four arcs arranged in two concentric groups. The inner and outer arcs have radii of 825 µm and 975 µm, respectively. This microstructure was fabricated using an SU-8 photoresist mold on a silicon wafer (Figure 2b). Liquid PDMS was prepared by blending the base and curing agent of a silicone elastomer (Dow Corning Sylgard 184, Midland, MI, USA) at a 10:1 weight ratio. Sugar particles with a crystal size of approximately 600 μm were then dispersed in the liquid PDMS at a 1:10 sugar-to-PDMS weight ratio. After 10 min of manual stirring, the PDMS-sugar mixture was evacuated for 20 min to remove trapped air bubbles. The mixture was then poured onto the microstructured mold to form a 1 mm thick layer (Figure 2c). A secondary evacuation process was performed to further degas the liquid PDMS. The mixture was subsequently cured in an oven at 60 °C for three hours (Figure 2d). Finally, the solid PDMS layer was peeled from the silicon mold (Figure 2e).

**Figure 3:**
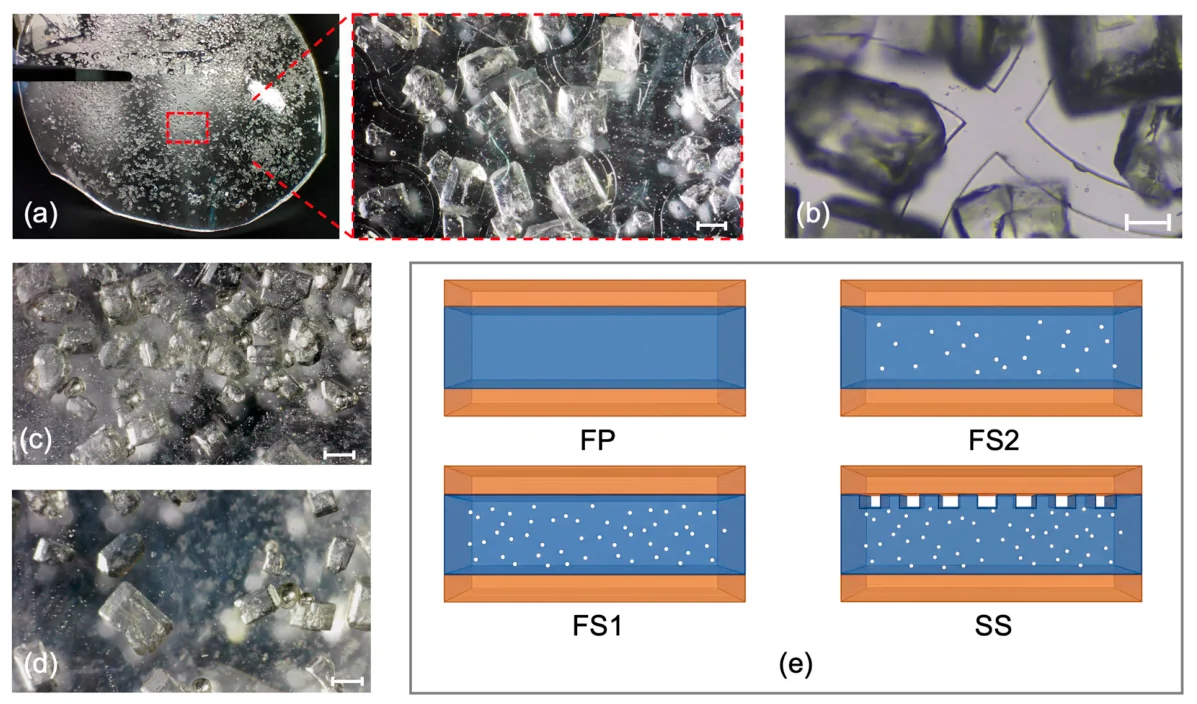
(**a**) Photograph and zoomed-in image (scale: 600 μm), and (**b**) microscopic image of the SS sensor (scale: 200 μm); zoomed-in image of the (**c**) FS1 sensor (scale: 600 μm) and (**d**) FS2 sensor (scale: 600 μm); (**e**) schematic illustration of FP layer, FS1 layer, FS2 layer, and SS layer, respectively.

**Figure 4:**
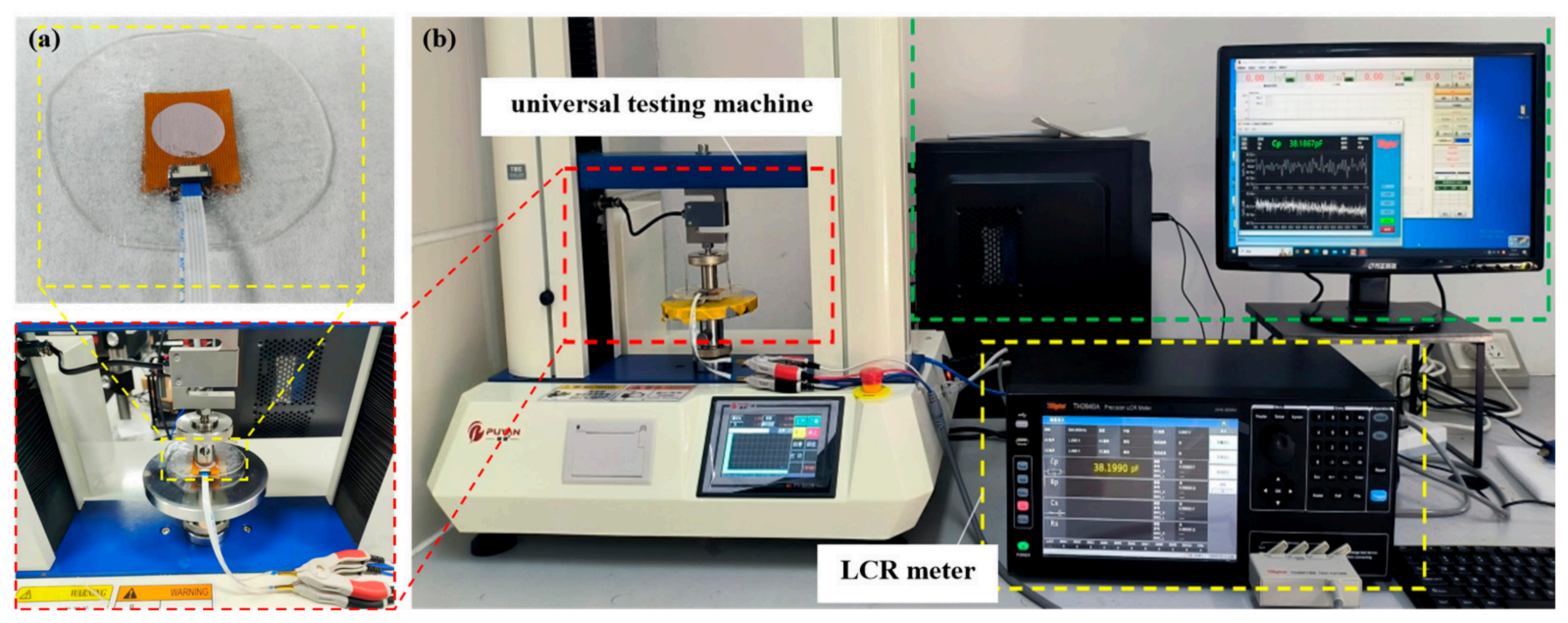
Photograph of (**a**) final sensor and (**b**) experimental setup.

Optical microscopy confirmed that the sugar particles were well-distributed within the cured PDMS layer (Figure 3a) and revealed the detailed morphologies of both the particles and the serpentine microstructure (Figure 3b). This composite layer, featuring both the serpentine pattern and sugar particles, was designated as the SS layer. For comparison, flat layers of the sugar-PDMS mixture were fabricated at weight ratios of 1:10 and 1:20, designated as FS1 and FS2, respectively (Figure 3c,d). A flat, pure PDMS layer of identical dimensions was also fabricated as a control (designated as the FP layer). The sensors with different functional layers are illustrated in Figure 3e. To assemble the pressure sensors, each dielectric functional layer was integrated with electrodes. Specifically, the functional layer was sandwiched between two polyimide (PI) films featuring 25 mm diameter circular copper pads (Figure 4a). The sensor was evaluated using a universal testing machine coupled with an LCR meter (Figure 4b). The testing machine applied force through a 20 mm compression cylinder, while the LCR meter recorded the relative change in capacitance (Δ*C*/*C*_0_), where *C*_0_ is the initial capacitance, and Δ*C* is the change in capacitance.

## 3. Results and Discussion

### 3.1. Limit of Detection and Resolution Testing

**Figure 5:**
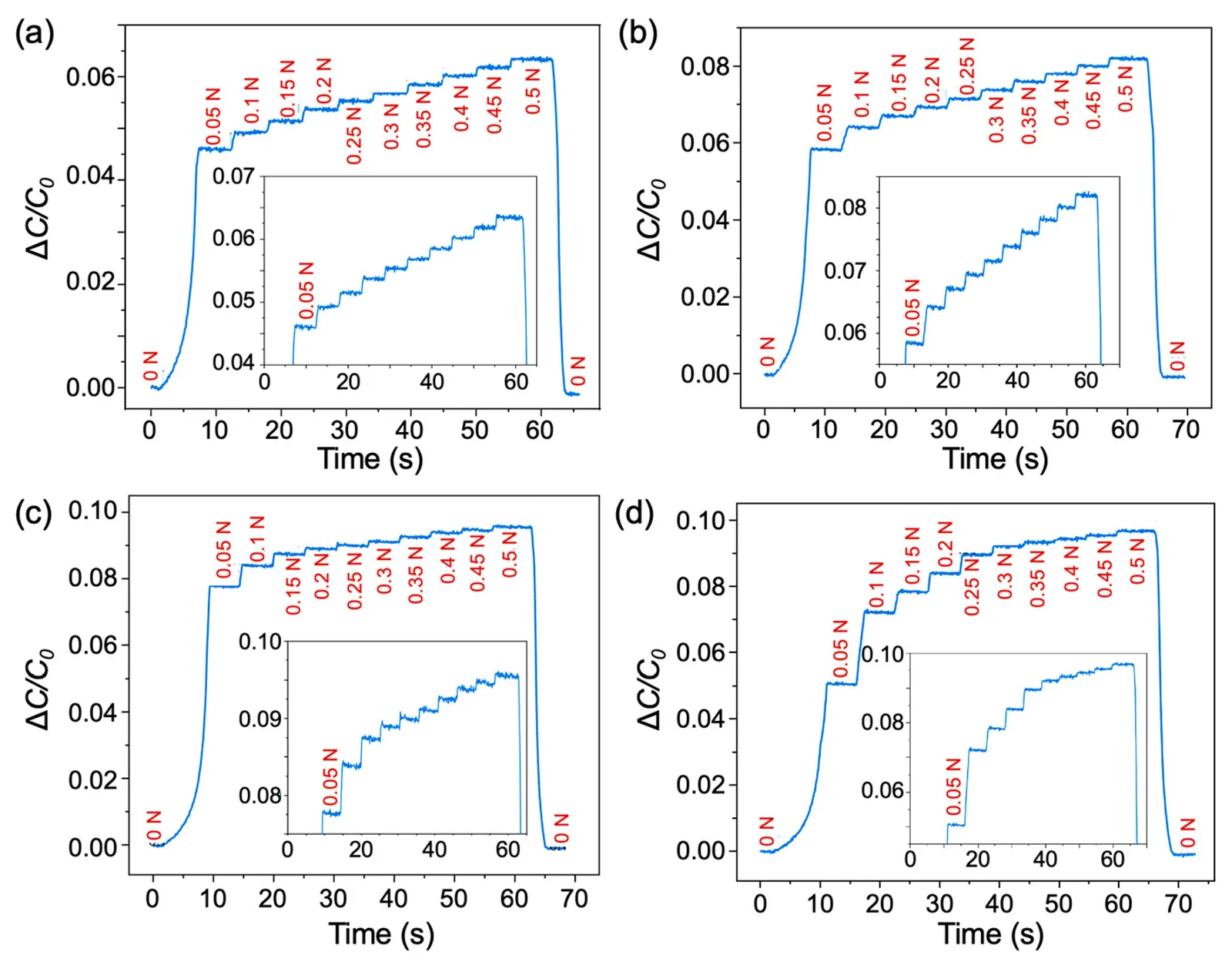
Step response of the (**a**) FP, (**b**) FS2, (**c**) FS1, and (**d**) SS sensors.

The limit of detection of the sensor was first investigated by evaluating Δ*C*/*C*_0_ at an applied force of 0.05 N, which corresponds to the lower limit of the universal testing machine. As shown in Figure 5, the capacitance between the electrode pads of the sensor exhibited a sudden increase of 4.6%, 5.8%, 7.8%, and 5.0% when the lower limit pressure force of 0.05 N was normally applied to the sensor with functional layers of FP, FS2, FS1, and SS, respectively. Evidently, all four sensors can detect a force as low as 0.05 N. When the applied force was doubled to 0.1 N, the capacitance change increased by only a fraction of that amount. This occurred because the initial capacitance (C_0_) was measured with no contact between the compression cylinder and the sensor, whereas the applied force induced initial contact in addition to the subsequent compressive deformation. The relative increase in capacitance change for the sensors with different functional layers was calculated as 8.7% (from 4.6% to 5.0%) for FP, 12.1% (from 5.8% to 6.5%) for FS2, 7.7% (from 7.8% to 8.4%) for FS1, and 42.0% (from 5.0% to 7.1%) for SS, indicating that the SS sensor exhibits the highest sensitivity to compressive force within the low-force range of 0–0.1 N.

**Figure 6:**
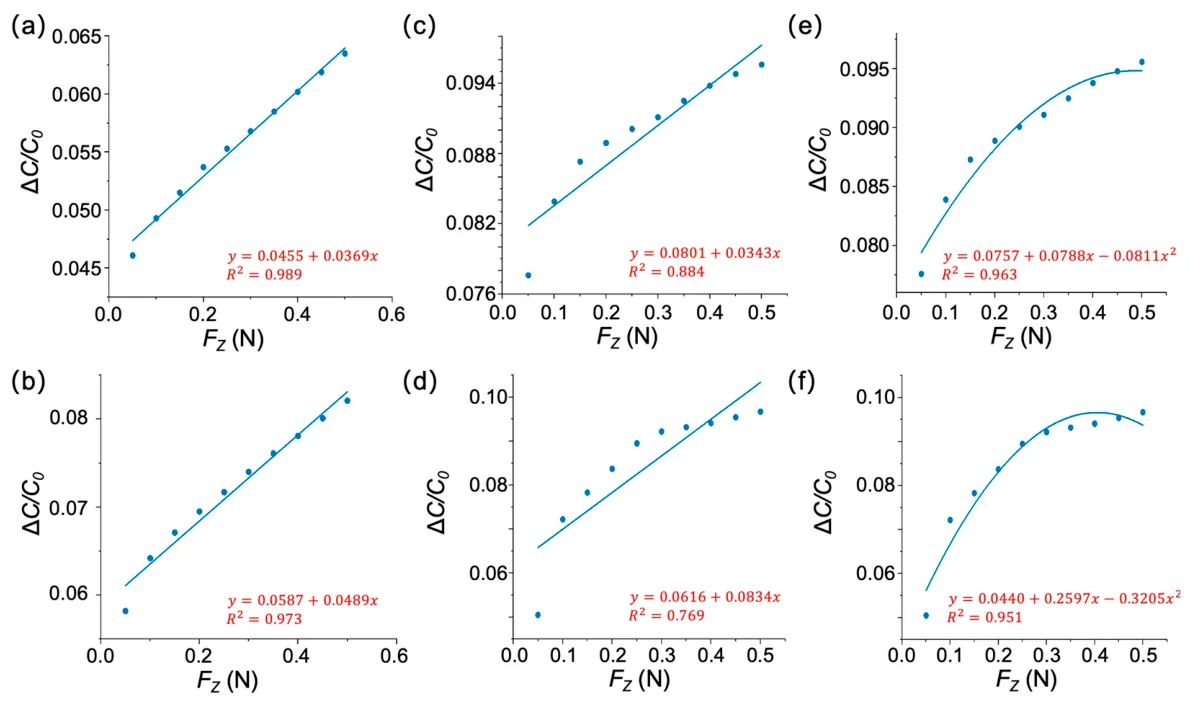
Linear fitting for (**a**) FP, (**b**) FS2, (**c**) FS1, and (**d**) SS sensors; and polynomial fitting for (**e**) FS1 and (**f**) SS sensors.

Next, the sensor’s resolution was evaluated by gradually increasing the force to 0.5 N in 0.05 N increments. A clear step-like response was observed from all sensors, indicating that the sensor resolution can be as low as 0.05 N over the 0–0.5 N range. The sensitivity of the sensors in this narrow force range was then analyzed by fitting the capacitance change to the applied force using a linear relation, $\Delta C/C_{0}\;=\;S\times F+M$, where *F* is the applied force, *S* is the sensitivity defined as the relative change in capacitance per Newton of force increment, and *M* is a constant. As shown in Figure 6a–d, the sensitivity is calculated as 0.04 N^−1^, 0.03 N^−1^, 0.05 N^−1^, and 0.08 N^−1^ for the FP, FS2, FS1, and SS sensors, respectively, while the corresponding determination coefficients (*R*^2^) are calculated as 0.99, 0.97, 0.88, and 0.77. This indicates that the sensing follows a strong linear relationship for the FP and FS2 sensors, but a poor linear relationship for the FS1 and SS sensors. Therefore, a second-order polynomial model, $y=ax^{2}+bx+c$, was fitted to the latter two sensors, as shown in Figure 6e,f, where *y* is the relative change in capacitance and *x* is the magnitude of the applied force. The fitting coefficients and corresponding determination coefficients are provided in the figure insets. Here, the *R*^2^ for both the FS1 and SS sensors is above 0.95, indicating that the polynomial model accurately captures the relationship between the relative change in capacitance and the magnitude of the applied force.

### 3.2. Sensitivity Measurement

**Figure 7:**
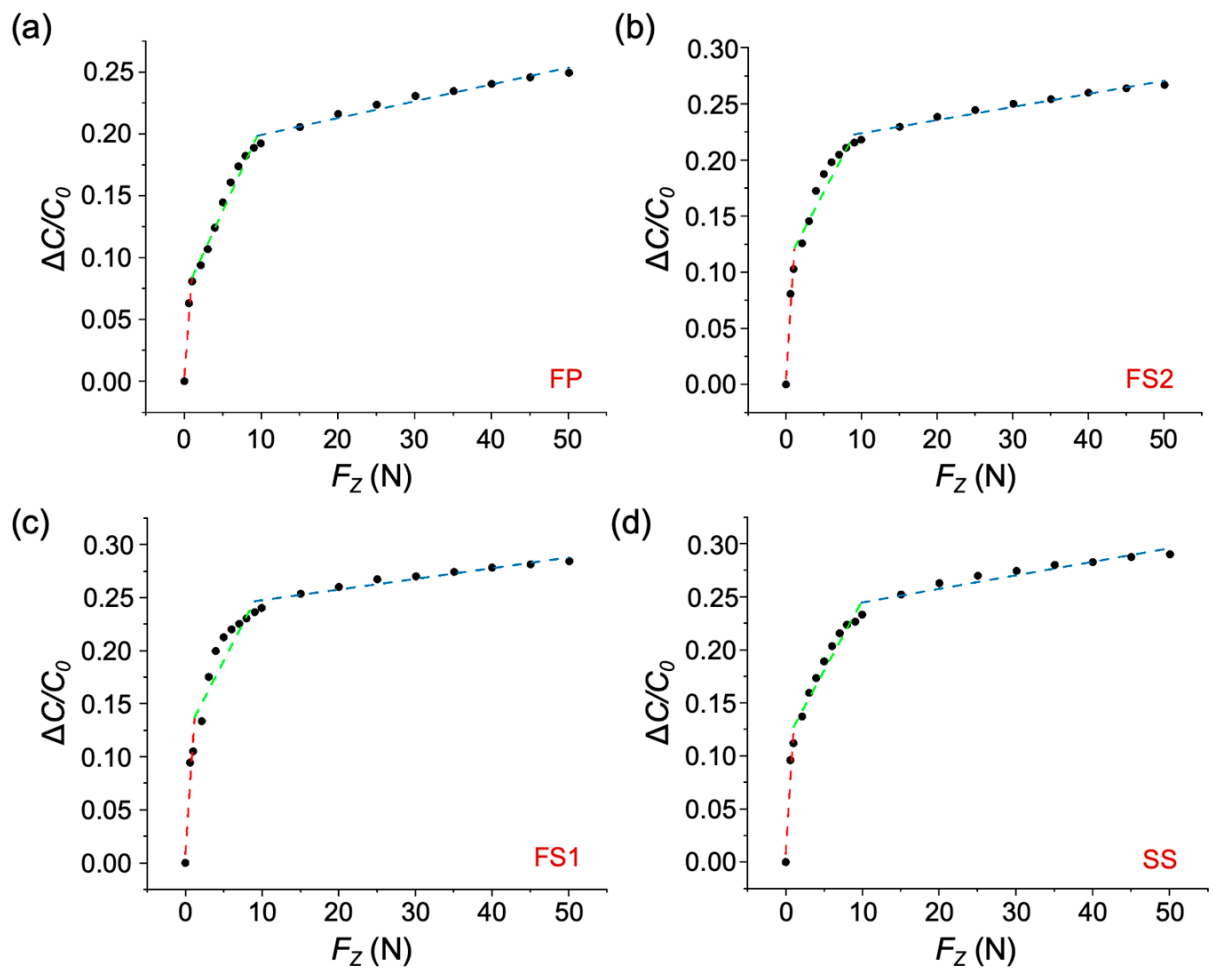
Measured relative change in capacitance (black dots) of the (**a**) FP, (**b**) FS2, (**c**) FS1, and (**d**) SS sensors, and the corresponding fitting curve (dashed line).

The sensor’s response to a broad range of normal pressure forces, from 0 to 50 N, was subsequently investigated. The relative capacitance change (Δ*C*/*C*_0_) for different sensors is plotted as black dots in Figure 7. Although capacitance increased with the applied force magnitude for all sensors, the rate of increase was highest in the 0–1 N range, decreased from 1 N to 10 N, and dropped further in the 10–50 N range. This phenomenon can primarily be attributed to the nonlinear behavior of the intermediate-layer material: under zero pressure, the molecular chains in the PDMS are relaxed, and the microstructure remains undeformed. Low pressure causes the molecular chains to transition from a relaxed to a compressed state. Deformation also begins to occur at the corner points of the microstructure in the functional layer, leading to a dramatic change in capacitance and high sensitivity. Under high pressure, the molecular chains of the internal material become compressed, exhibiting increasing resistance to deformation. Furthermore, the microstructural deformation transitions from a “local point contact” to a “full area compression” state, making further compression increasingly difficult; consequently, the capacitance value tends to saturate with minimal change, resulting in decreased sensor sensitivity.

Subsequently, the sensitivity of each sensor was evaluated by fitting the measured results to a linear function across different force ranges; the red, green, and blue dashed lines represent the fitting curves for the 0–1 N, 1–10 N, and 10–50 N ranges, respectively. The relative change in capacitance per unit force, *S*_F_ (the slope of the linear fit), for each sensor within each range is summarized in Table 1. Additionally, the sensitivity per unit pressure, *S*_P_ = *S*_F_/(π*r*^2^), is also reported in Table 1, where *r* = 10 mm is the radius of the compression cylinder. In the 0–1 N force range, a sensitivity (*S*_P_) of 2.55 × 10^−2^ kPa^−1^, 3.25 × 10^−2^ kPa^−1^, 3.33 × 10^−2^ kPa^−1^, and 3.55 × 10^−2^ kPa^−1^ was obtained for the FP, FS1, FS2, and SS sensors, respectively. Compared with the FP sensor, the FS2 sensor exhibited up to a 38.5% increase in sensitivity, demonstrating that the hybrid microstructured layer effectively improved sensitivity within this range. For the 1–10 N force range, the sensitivities of the aforementioned sensors are 4.2 × 10^−3^ kPa^−1^, 4.0 × 10^−3^ kPa^−1^, 4.3 × 10^−3^ kPa^−1^, and 4.1 × 10^−3^ kPa^−1^, respectively. The sensitivity varies by only 7.5%, indicating that the microstructure has a negligible impact on sensitivity in this range. Finally, for the 10–50 N force range, the sensitivity is approximately two orders of magnitude smaller than that in the 0–1 N range, indicating that this device is unsuitable for sensing forces above 10 N.

### 3.3. Repeatability and Hysteresis Testing

**Figure 8:**
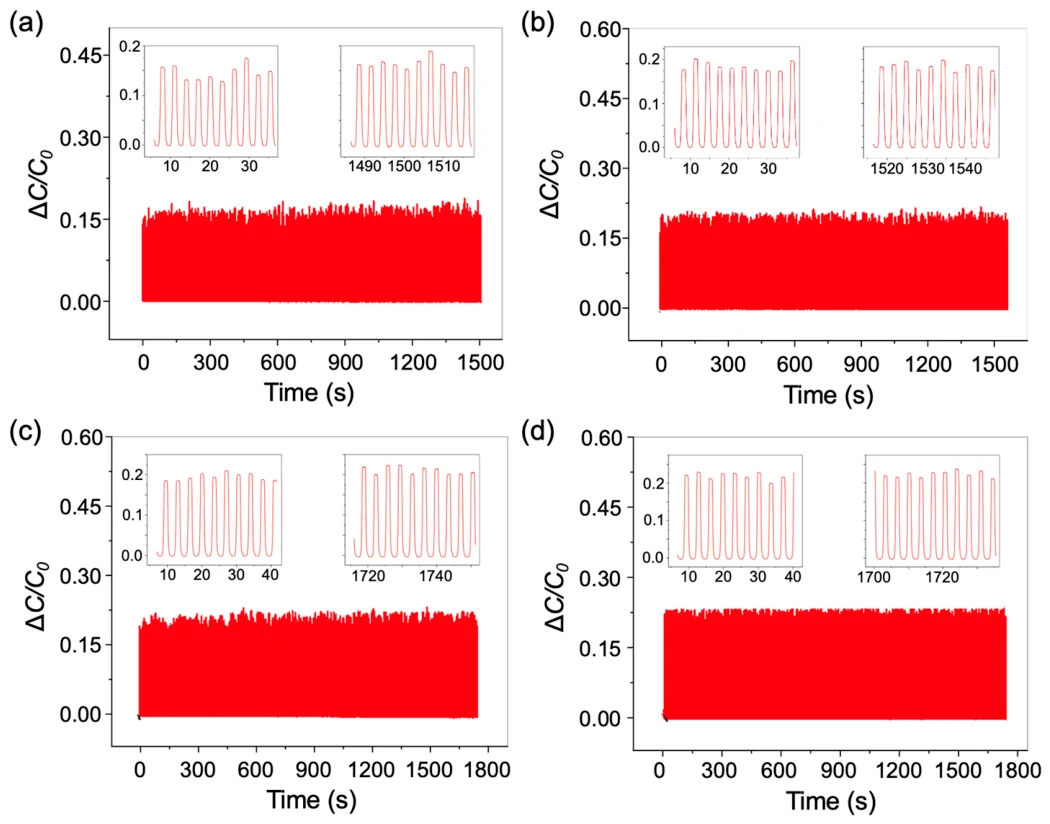
Measured repeatability of the (**a**) FP, (**b**) FS2, (**c**) FS1, and (**d**) SS sensors.

To evaluate sensor repeatability, cyclic loading–unloading tests were performed using an electronic universal testing machine. A normal force ranging from 0 to 5 N was repeatedly applied to the sensor for over 500 consecutive cycles under stable ambient laboratory conditions (25 ± 2 °C). During the measurements, the LCR meter continuously recorded the capacitance at a sampling rate of 100 kHz. The measured relative capacitance change over time was plotted in Figure 8. Repeatability was then quantified by evaluating the variance of the capacitance change under maximum load, which was 1.96 × 10^−4^, 8.25 × 10^−6^, 4.21 × 10^−6^, and 1.24 × 10^−6^ for the sensors with functional layers of FP, FS1, FS2, and SS, respectively. These results indicate that the sensor’s repeatability is significantly improved by integrating the hybrid-structured functional layer.

**Figure 9:**
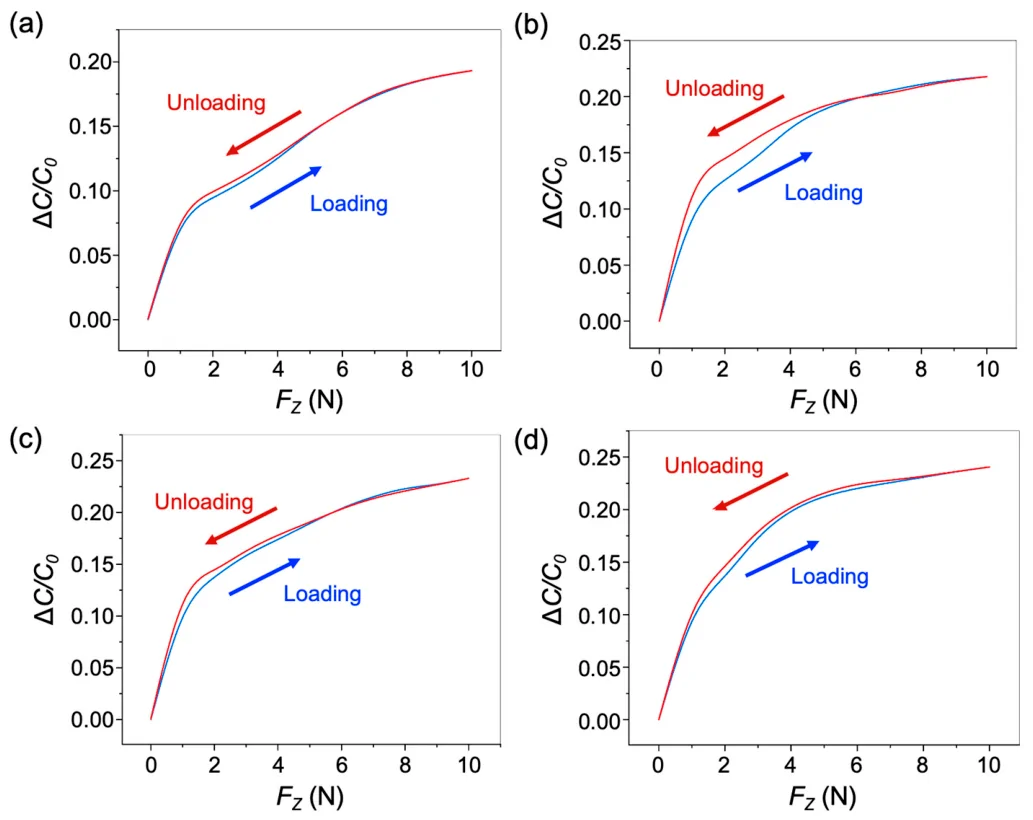
Measured hysteresis of the (**a**) FP, (**b**) FS2, (**c**) FS1, and (**d**) SS sensors.

Hysteresis, another important parameter of the pressure sensor, quantifies the difference between the sensor’s output readings during the loading and unloading phases. Here, the universal testing machine was set to apply a force of 0–10 N to the sensors. The force was gradually applied to the sensor, then slowly unloaded, with the capacitance recorded in real time. Figure 9a–d show the hysteresis curves of the sensors with functional layers of FP, FS2, FS1, and SS, respectively. The hysteresis curves follow the blue lines during loading and the red lines during unloading. In all cases, the capacitance returned to its original value after the loading–unloading cycle. However, the blue and red lines did not fully overlap, indicating hysteresis in the sensors. This phenomenon arises because, during the unloading stage, the sensor retains residual deformation at a higher force than in the previous instance. Consequently, the loading curve lies below the unloading curve on the hysteresis plot.

Furthermore, the blue and red lines of the hysteresis curve almost overlap in the force ranges of 0–1 N and 5–10 N, while an observable difference exists within the force range of 1–5 N. The hysteresis value, defined as the maximum of (∆*C*_unloading_ − ∆*C*_loading_)/*C*_0_ during the loading–unloading process, was measured as 0.6%, 2.3%, 1.3%, and 0.9% at applied forces of 1.7 N, 1.4 N, 1.3 N, and 1.8 N for the sensors with functional layers of FP, FS2, FS1, and SS, respectively. The increased hysteresis observed in both the FS2 and FS1 sensors is attributed to the non-vertical pressure distribution acting on the sugar microstructure, which induces a slight tangential sliding that requires overcoming static friction. When the pressure is released, the contact area decreases, and the stored elastic potential energy is released, causing the microstructure to restore instantaneously. Overall, the four functional layers exhibit different hysteresis characteristics due to their distinct structural configurations. The FP sensor, consisting of a homogeneous PDMS dielectric layer, exhibits the lowest hysteresis, whereas incorporating sugar particles into the FS1 and FS2 sensors increases hysteresis; this may be associated with additional viscoelastic deformation and interfacial mechanical interactions within the composite dielectric layer. In comparison, the SS sensor exhibits lower hysteresis than both FS1 and FS2, suggesting that the serpentine microstructure helps stabilize the deformation process and partially mitigates the hysteresis introduced by particle embedding. Further mechanical characterization will be conducted in future work to verify this mechanism.

### 3.4. Response Time and Relaxation Time Testing

**Figure 10:**
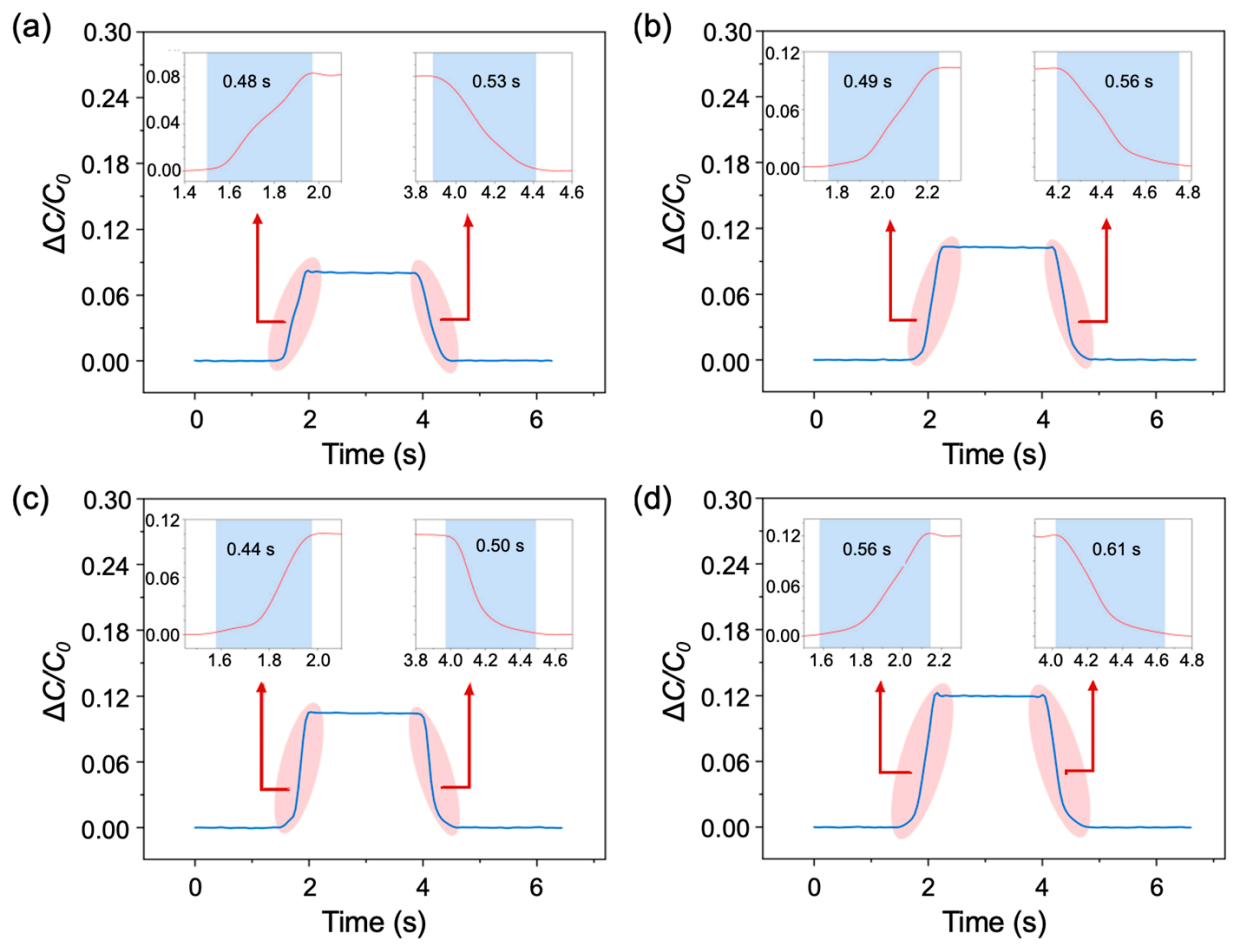
Measured response time and relaxation time of the (**a**) FP, (**b**) FS2, (**c**) FS1, and (**d**) SS sensors.

For the dynamic response measurement, the sensor was loaded from 0 to 1 N at a relatively fast rate of 3 mm/s, held at the target force for 2 s, and then unloaded at the same rate. The capacitance variation was continuously monitored throughout the loading and unloading processes. The response time was defined as the time required for the capacitance signal to increase from 10% to 90% of its steady-state value after loading, whereas the relaxation time was defined as the time required for the signal to decrease from 90% to 10% of its steady-state value after unloading. The measured Δ*C*/*C*_0_, as shown in Figure 10, increased from 0 to 8.1%, 10.1%, 10.3%, and 12.0% when the 1 N force was applied to the FP, FS1, FS2, and SS sensors, respectively; all signals returned to zero after the force was unloaded. Meanwhile, the response times of the FP, FS1, FS2, and SS sensors were recorded as 0.48 s, 0.49 s, 0.44 s, and 0.56 s, respectively, while their relaxation times were 0.53 s, 0.56 s, 0.5 s, and 0.61 s, respectively. The response time is relatively slow compared with sensors with millisecond-level dynamic performance, a limitation primarily due to the viscoelastic characteristics of the hybrid dielectric layer. First, the PDMS matrix inherently exhibits time-dependent viscoelastic deformation and recovery. Second, the embedded sugar particles introduce local mechanical constraints that increase the redistribution of internal stresses during loading and unloading. Third, the serpentine microstructured surface undergoes gradual deformation and recovery rather than instantaneous elastic rebound, further prolonging the stabilization process. These combined effects improve pressure sensitivity and hysteresis performance but inevitably sacrifice response speed.

### 3.5. Non-Contact Mode Measurement

**Figure 11:**
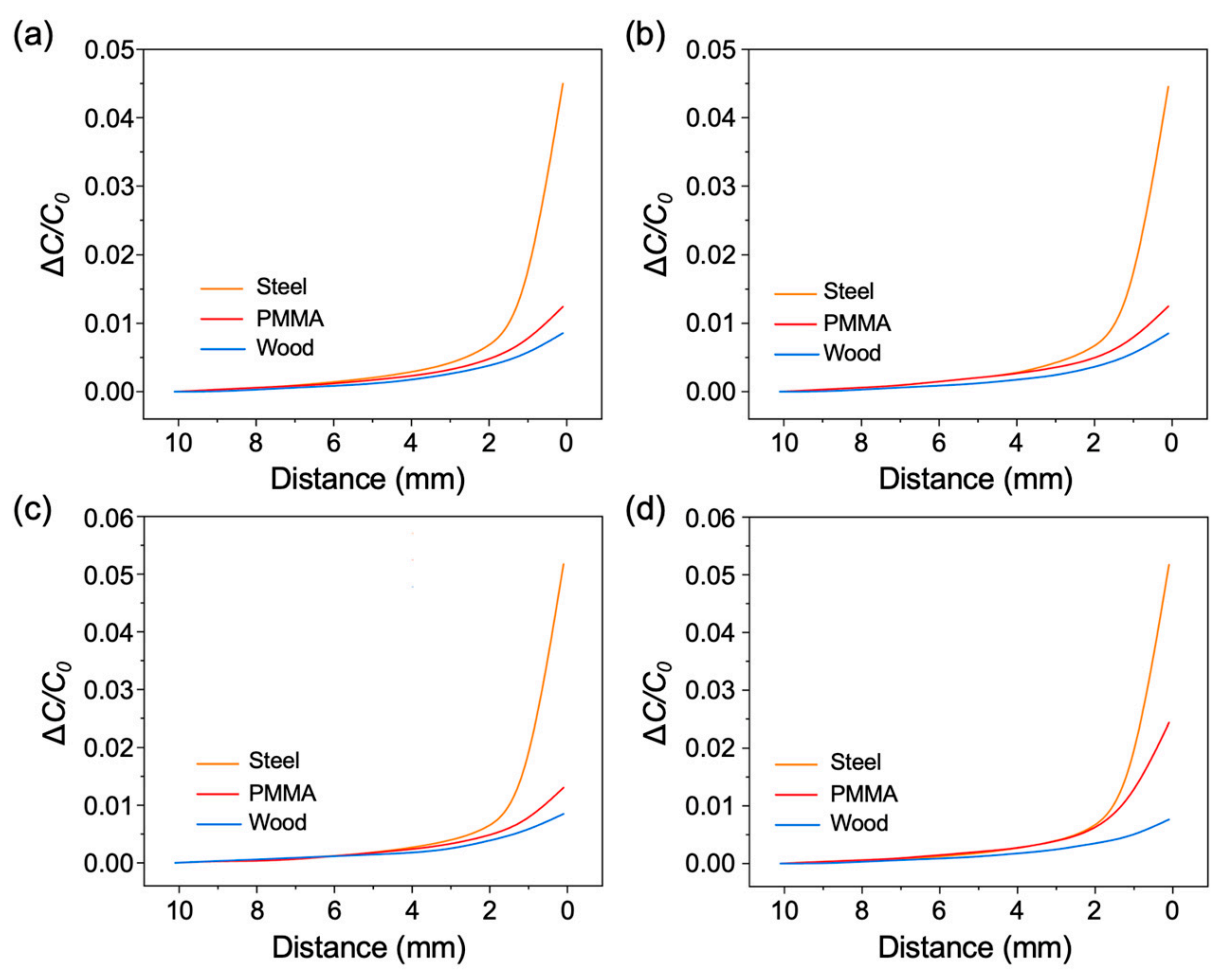
Non-contact distance monitoring using the (**a**) FP, (**b**) FS2, (**c**) FS1, and (**d**) SS sensors.

**Figure 12:**
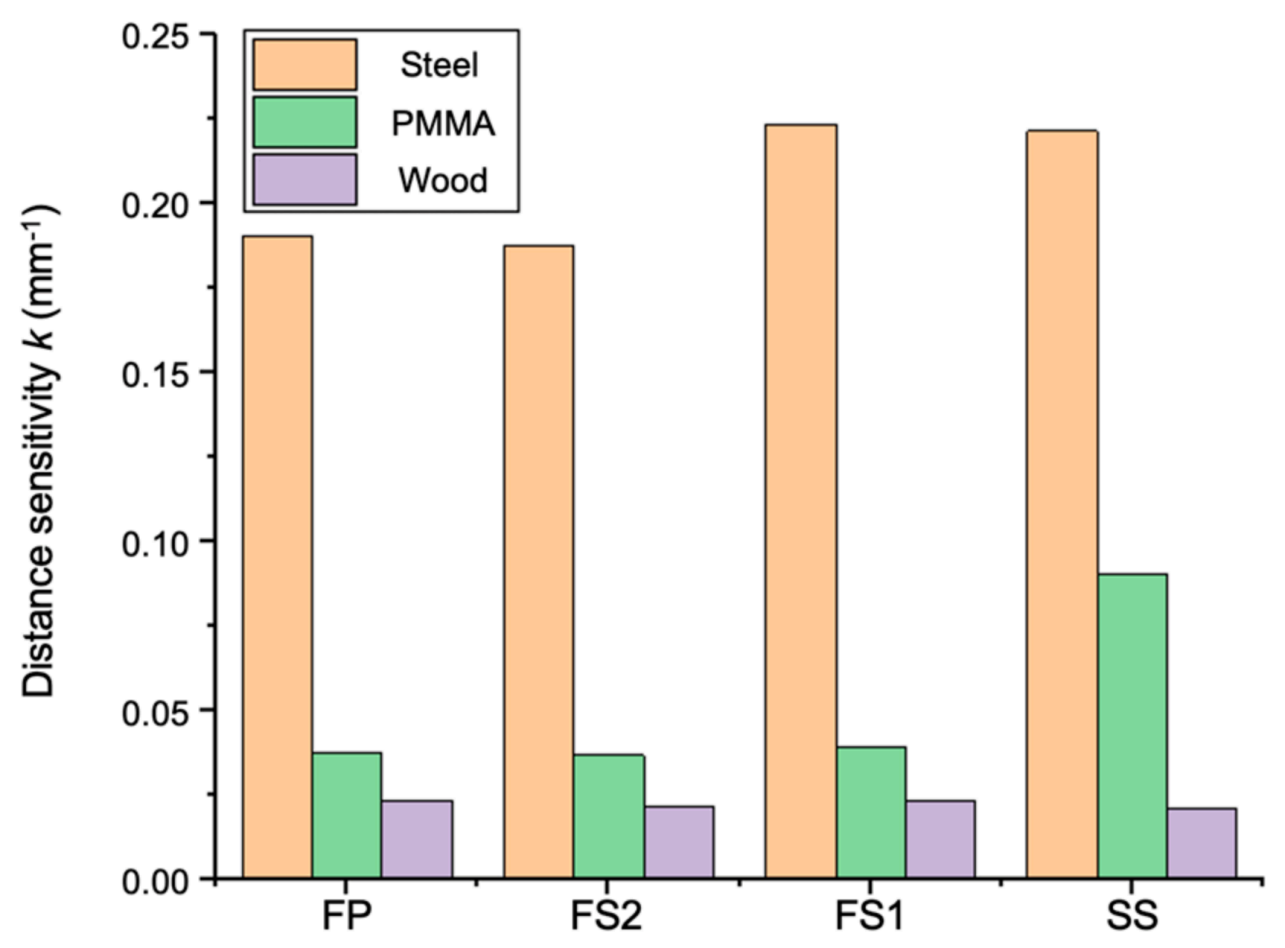
Material identification using different sensors.

Inspired by the sudden increase in the sensor signal when the compression cylinder initially made contact, we speculated that the capacitance would also change when the steel cylinder approached the sensor without making contact. We measured the change in capacitance while gradually lowering the cylinder toward the sensor from an initial distance of 10 mm, as shown in Figure 11. For comparison, we also performed these measurements with the cylinder attached to a 2 mm-thick layer of polymethyl methacrylate (PMMA) and a 2 mm-thick layer of spruce wood. When the different cylinders were positioned near the sensors at a distance of 10 mm, we observed a small increase in capacitance across all cases, provided the distance exceeded 2 mm, indicating low sensitivity to distance changes in this outer range. However, at distances below 2 mm, the capacitance increased significantly in all cases. The rates of increase as the different cylinders approached the sensors were calculated, as shown in Figure 12. The rate of increase was highest when the steel cylinder approached the sensor, calculated as 0.018 mm^−1^, 0.018 mm^−1^, 0.023 mm^−1^, and 0.023 mm^−1^ for the FP, FS2, FS1, and SS sensors, respectively. Consequently, the sensors can be used to monitor changes in proximity. In addition, the rate of capacitance change was much lower when the cylinder was attached to PMMA and lowest when attached to wood. This is because steel has an approximately infinite permittivity, while PMMA and spruce wood have relative permittivities of about 3.7 and 2.1 under DC conditions, respectively. Therefore, the sensors can also be used to distinguish different materials under non-contact conditions.

## 4. Conclusions

In conclusion, a multifunctional sensor was developed for pressure sensing, distance monitoring, and material identification. The sensor utilizes a hybrid microstructured functional layer, which significantly improved its pressure-sensing performance, achieving a high sensitivity of 3.55 × 10^−2^ kPa^−1^ at applied forces up to 1 N, a low hysteresis of 0.9%, and robust repeatability with an ultra-low variance of 1.24 × 10^−6^. In addition, the study robustly demonstrated that the sensor can monitor proximity to an object with a sensitivity of 0.023 mm^−1^ and distinguish between materials with different permittivities.

Furthermore, these combined dual-mode (contact and non-contact) capabilities make the device highly promising for next-generation flexible electronics. Specifically, its high sensitivity at low pressure makes it suitable for wearable health-monitoring patches, while its non-contact distance and material-profiling capabilities can be leveraged in proximity-sensing emergency controls, automated industrial sorting systems, and context-aware smart city infrastructure. However, the slow response time in the contact pressure-sensing mode limits the sensor’s suitability for applications requiring rapid dynamic responses. Therefore, the present sensor is more suitable for quasi-static or low-frequency applications, such as wearable health monitoring, proximity sensing, material identification, and slow robotic tactile interaction. Future work will focus on optimizing the dielectric material and microstructure to shorten the response time while maintaining the sensing performance.

## Figures and Tables

**Table 1:** Calculated sensitivity of different pressure sensors.

Sensors Force (N)		0–1	1–10	10–50
FP	*S*_F_ (×10^−2^ N^−1^)	8.12	1.34	0.13
	*S*_P_ (×10^−2^ kPa^−1^)	2.55	0.42	0.04
FS2	*S*_F_ (×10^−2^ N^−1^)	10.35	1.27	0.13
	*S*_P_ (×10^−2^ kPa^−1^)	3.25	0.40	0.04
FS1	*S*_F_ (×10^−2^ N^−1^)	10.60	1.37	0.10
	*S*_P_ (×10^−2^ kPa^−1^)	3.33	0.43	0.03
SS	*S*_F_ (×10^−2^ N^−1^)	11.30	1.31	0.13
	*S*_P_ (×10^−2^ kPa^−1^)	3.55	0.41	0.04

## Data Availability

Raw data presented in this study are available on request from the corresponding author.

## Abbreviations

The following abbreviations are used in this manuscript:

PDMS	polydimethylsiloxane
PMMA	polymethyl methacrylate
PI	polyimide
